# Reframing nonsense variants in genodermatoses through personalized molecular rescue

**DOI:** 10.1016/j.omtn.2026.103159

**Published:** 2026-11-09

**Authors:** Yue Li, Ming Li

**Affiliations:** 1 Department of Dermatology, Children’s Hospital of Fudan University, National Children’s Medical Center, Shanghai 201102, China

## Main text

Nonsense variants offer a promising therapeutic target in genodermatoses because premature translation termination can potentially be bypassed. A premature termination codon (PTC) can produce a truncated protein and trigger nonsense-mediated mRNA decay (NMD), reducing the transcript available for protein synthesis. Even partial restoration may be biologically meaningful. In *COL17A1*-related junctional epidermolysis bullosa (JEB), genotype-phenotype studies have shown that C17 expression at only approximately 12%–14% of normal levels can ameliorate the disease phenotype.^1^ These observations support protein rescue as a therapeutic goal, although they do not establish a universal efficacy threshold. A small amount of correctly localized adhesion protein may be more useful than a larger amount that fails to assemble into the relevant structural complex.

The relevance of nonsense suppression extends across several genodermatoses. Approximately 20% of *COL17A1*-related JEB cases involve nonsense variants, and *LAMB3*-associated severe JEB is enriched for this variant class.^2^^,^^3^ PTC-causing variants are also estimated to occur in at least 15%–20% of patients with recessive dystrophic epidermolysis bullosa (RDEB), caused by *COL7A1* deficiency.^4^ In Nagashima-type palmoplantar keratoderma (NPPK), the *SERPINB7* founder variant c.796C>T (p.Arg266∗) is particularly frequent in East Asian populations.^5^^,^^6^ Gentamicin has restored functional laminin-332 in experimental *LAMB3*-associated JEB 3D skin models, while a randomized, double-blind, vehicle-controlled NPPK study demonstrated improvements in hyperkeratosis and malodor.^3^^,^^6^ These findings establish complementary experimental and clinical precedents for targeting premature termination.

In this issue of *Molecular Therapy Nucleic Acids*, Tan et al. demonstrated how to select readthrough therapies suitable for individual *COL17A1* genotypes.^2^ Screening 10 translational readthrough-inducing drugs (TRIDs) in patient-derived keratinocytes carrying six nonsense variants revealed substantial response heterogeneity. W464∗ responded strongly to aminoglycosides, R688∗ weakly, and R1226∗ showed no detectable response. R145∗ responded to ataluren (PTC124), whereas R169∗ was refractory to the tested compounds. All six variants generated UGA codons, demonstrating that stop-codon identity alone cannot guide treatment selection. Differences in transcript abundance and residual C17 expression further indicate that available mRNA substrate and baseline protein expression must be considered. Importantly, R145∗ and G803∗ cells carried a second pathogenic allele, so their responses cannot be attributed exclusively to the nonsense variant.

A notable contribution is the demonstration that N-oxalylglycine (NOG) can enhance aminoglycoside-based rescue in selected JEB-C17 backgrounds.^2^ In W464∗ keratinocytes, adding NOG to gentamicin, paromomycin, and CC-90009 increased C17 by approximately 70% relative to the combination without NOG, reaching approximately 15% of normal keratinocyte levels. No impairment of viability or scratch-wound closure was detected under the tested conditions. NOG also enhanced C17 expression in G803∗ cells when combined with gentamicin and CC-90009, but provided no benefit in R145∗ and did not rescue R1226∗. However, PTC124 alone increased total C17 in R145∗ cells from 25.6% to 52% of normal, without further benefit from the tested adjuncts. Because substantial protein was present before treatment, this endpoint should not be interpreted as a direct measurement of nonsense-allele readthrough efficiency. Together, these results show that combination therapy must be evaluated experimentally for each genotype.

Mechanistic distinctions are essential when interpreting these combinations (Figure 1). Gentamicin and paromomycin promote ribosomal readthrough; CC-90009 is an eRF3a/GSPT1 degrader that can reduce translation termination efficiency and suppress NMD.^2^ NOG reduces eRF1 hydroxylation and has been investigated as a modulator of termination and NMD.^2^ These adjuncts can enhance readthrough through mechanisms distinct from the aminoglycoside effects on ribosomal decoding. In the COL17A1 study, increased mRNA levels during combined treatment suggest improved transcript stability, although the mechanism remains unclear.

**Figure 1:**
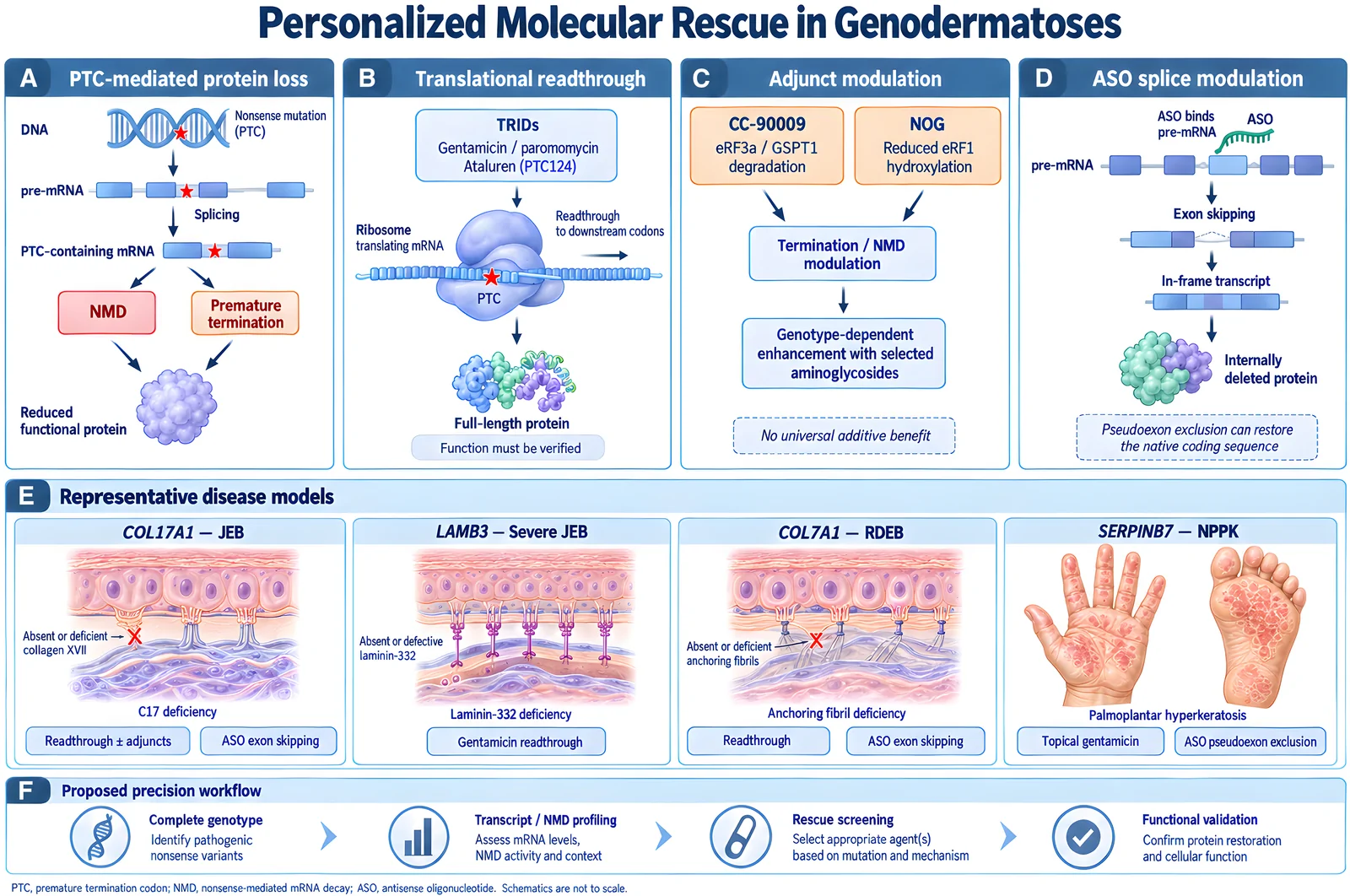
Personalized translational and splicing rescue in genodermatoses (A) A PTC can cause premature translation termination and NMD, reducing functional protein. (B) TRIDs, exemplified by gentamicin, paromomycin, and ataluren, promote translation beyond the PTC. (C) CC-90009 and NOG are shown separately as release-factor/NMD-modulating adjuncts. CC-90009 promotes eRF3a/GSPT1 degradation, whereas NOG reduces eRF1 hydroxylation. (D) Splice-modulating ASOs can exclude a suitable mutation-containing exon or an aberrant pseudoexon. Exon skipping produces an internally deleted protein; pseudoexon exclusion may restore the native coding sequence. (E) Representative disease models comprise *COL17A1*-related JEB, *LAMB3*-associated severe JEB, *COL7A1*-related RDEB, and *SERPINB7*-related NPPK. (F) Proposed selection workflow integrating the complete genotype, transcript/NMD assessment, rescue screening, and tissue-level functional validation.

Splice-modulating antisense oligonucleotides (ASOs) provide another route to protein restoration. Unlike readthrough, which acts during translation, ASOs bind pre-mRNA and alter RNA processing. Their potential targets extend beyond nonsense substitutions to selected frameshift, splice-site, and deep-intronic variants, provided that an appropriate splice intervention exists. ASO-mediated exon 7 skipping restored an in-frame *COL17A1* transcript and C17 localization in keratinocytes and 3D skin equivalents carrying c.380-1G>A.^7^ In RDEB, *COL7A1* exon 105 skipping restored C7 production in patient-derived cells and *de novo* expression in human skin grafts.^8^ ASOs also corrected pseudoexon inclusion caused by the deep-intronic *SERPINB7* variant c.745-553T>G.^9^ Table 1 summarizes representative strategies and their current levels of evidence.

**Table 1:** Representative translational and splicing rescue strategies in genodermatoses

Disease and gene	Representative defect	Strategy and action	Evidence	Main constraints
*COL17A1*-related JEB	W464⁎, R145⁎, G803⁎, R1226⁎; splice variant c.380-1G>A	TRIDs promote translational readthrough; CC-90009/NOG is distinct adjunct. ASOs can induce exon 7 skipping	W464⁎: ∼15% of normal C17 with gentamicin + paromomycin + CC-90009 + NOG. R145⁎: total C17 25.6% → 52% with PTC124.^2^ Exon 7 skipping restored C17 in cell/3D models^7^	variant and allele dependence; incomplete rescue; NMD/termination effects need resolution; suitable exon and delivery required
*LAMB3*-associated severe JEB	selected nonsense variants, including R635⁎ and C290⁎	gentamicin-mediated translational readthrough	full-length laminin β3 restored from eight tested nonsense variants; laminin-332 assembly and deposition recovered in experimental models^3^	preclinical evidence; variable rescue and aminoglycoside toxicity
*COL7A1*-related RDEB	PTC-causing variants; selected exon 105-associated variants	readthrough for responsive nonsense variants; ASO-mediated exon skipping for suitable exonic defects	small-molecule readthrough examined in patient keratinocytes.^4^ Exon 105 skipping restored C7 in patient cells and human skin grafts^8^	readthrough cannot restore a frameshift by bypass alone; exon skipping requires preserved frame and C7 function; delivery/durability
*SERPINB7*-related NPPK	c.796C>T (p.Arg266⁎); c.745-553T>G (deep intronic)	topical gentamicin readthrough; ASO-mediated pseudoexon exclusion	readthrough studied clinical improvement in hyperkeratosis/malodor in a randomized trial.^6^ Pseudoexon inclusion corrected *in vitro*^9^	incomplete/transient benefit; erythema did not improve versus vehicle; ASO evidence remains preclinical

ASO, antisense oligonucleotide; C17, collagen XVII; C7, collagen VII; JEB, junctional epidermolysis bullosa; NMD, nonsense-mediated mRNA decay; NOG, N-oxalylglycine; NPPK, Nagashima-type palmoplantar keratoderma; PTC, premature termination codon; RDEB, recessive dystrophic epidermolysis bullosa; TRID, translational readthrough-inducing drug.

For early coding-region variants, removal of a short, dispensable exon may preserve most downstream protein domains and offer substantial rescue. However, N-terminal position alone does not predict ASO efficacy or functional benefit. Selection requires compatible exon phases, preservation of the reading frame, and retention of essential structural and interaction domains. Studies of C7 lacking exon 13 or exon 105 demonstrated retained function despite deletions near opposite ends of the protein.^10^ These findings favor exon-specific validation over a general preference for N-terminal targets. Splice correction that excludes an aberrant pseudoexon may preserve the native coding sequence, whereas therapeutic exon skipping usually produces an internally deleted protein requiring independent functional assessment.

Both readthrough and splice modulation remain limited by incomplete and variable rescue. Readthrough depends on transcript availability, surrounding sequence, and the functional consequences of the amino acid inserted at the PTC.^6^ Increasing mRNA abundance cannot guarantee productive translation, as the refractory R1226∗ model illustrates. Aminoglycoside toxicity and the wider effects of release-factor or NMD modulation also constrain prolonged exposure.^2^ ASO activity depends on target accessibility, intracellular delivery, and splice specificity; unintended splicing and insufficient skin penetration may limit benefit. Durability is likewise unresolved. In the NPPK trial, most participants relapsed after treatment withdrawal, supporting the need for sustained or repeated exposure.^6^ Short-term tolerability and protein recovery in cultured cells cannot establish long-term clinical efficacy or safety.

Tan et al. supported a treatment-development workflow that begins with the complete genotype and proceeds through transcript analysis, empirical drug selection, and functional validation.^2^ For variants poorly responsive to readthrough, a suitable splice window may justify ASO development. Future work should establish tissue deposition, mechanical adhesion, and durable rescue in skin models before clinical translation. The objective is to restore sufficient functional protein in the relevant tissue compartment to improve disease. Readthrough and ASOs should be viewed not as competing technologies but as complementary components of a personalized RNA-therapeutic toolbox. Such a strategy may ultimately transform nonsense suppression from a broadly defined therapeutic concept into a variant-specific, experimentally guided treatment pathway.

## Acknowledgments

This work was supported by grant from the National Natural Science Foundation of China (82473509).

## Author contributions

Y.L. and M.L. contributed to the conceptualization, design, and visualization of this commentary. Both authors participated in writing the original draft, reviewing, and editing the manuscript, and M.L. provided project administration and supervision.

## Declaration of interests

The authors declare no competing interests.

## Declaration of generative AI and AI-assisted technologies in the manuscript preparation process

During the preparation of this work, the authors used ChatGPT to check some parts of the text for spelling and grammar errors and to assist in increasing the resolution of the figure. After using this tool, the authors reviewed and edited the content as needed and take full responsibility for the content of the publication.
